# Correction effects of the ScoliOlogiC^® ^„Chêneau light" brace in patients with scoliosis

**DOI:** 10.1186/1748-7161-2-2

**Published:** 2007-01-26

**Authors:** Hans-Rudolf Weiss, Mario Werkmann, Carola Stephan

**Affiliations:** 1 Asklepios Katharina Schroth Spinal Deformities Rehabilitation Centre, Korczakstr. 2, D-55566 Bad Sobernheim, Germany; 2 Orthomed Scolicare, Orthopedic Technical Services, D-55566 Bad Sobernheim, Germany

## Abstract

**Background:**

Different bracing concepts are used today for the treatment of scoliosis. The plaster cast method worldwide seems to be the most practiced technique at the moment. CAD (Computer Aided Design) systems are on the market which allow brace adjustments without plaster. The latest development however, is the use of the ScoliOlogiC™ off the shelf system enabling the orthopaedic technician to construct a light brace for scoliosis correction from a variety of pattern specific shells to be connected to an anterior and a posterior upright. This „Chêneau light" brace, developed according to the Chêneau principle, promises a reduced impediment of quality of life in the brace. However, material reduction should not result in reduced effectiveness. Therefore the primary correction effect in the „Chêneau light" brace has been evaluated and compared with that of other braces used today.

**Methods:**

The correction effects of the first 81 patients (main diagnosis Adolescent Idiopathic Scoliosis (AIS) [n = 64] or Early Onset Scoliosis (EOS) [n = 15]), treated according to the principle of the „Chêneau light" brace were evaluated after an average treatment time of 6 weeks by a full-body X-ray made in the standing position whilst wearing the brace and compared with the last X-ray before bracing. The average curvature angle of the whole group was 35,6°, the average age was 12,9 years (SD 1,9), average Risser sign was 1,3 (SD 1,5), average Tanner rating 2,75 (SD 0,7).

**Results:**

The Cobb angle in the whole group was reduced by an average of 16,4°, which corresponds to a correction effect of 51%. The differences were highly significant in the T-test (T = 17,4; p < 0,001). The best correction effects reported in literature so far are about 40% in two different studies. The correction effect was highest in lumbar and thoracolumbar curve pattern (62 %; n = 18). In thoracic scoliosis the correction effect was 36 % (n = 41) and in double major curve pattern 50 % (n = 22). The correction effect correlated slightly negative with age (r = -0,24; p = 0,014), negatively with the Risser stage (-0,29; p = 0,0096) and correlated negatively with the Cobb angle measured before treatment (r = -0,43; p < 0,0001).

**Conclusion:**

The use of the „Chêneau light" brace leads to correction effects above average when compared to the correction effects of other braces described in literature. The reduction of material seems to affect the desired correction in a positive way.

## Background

The latest developments in the field of bracing, aim at improving specificity [1] and at a proper sagittal realignment [2].

Although the effect of brace treatment has been questioned [3] there is evidence that brace treatment can stop curvature progression [4-9], reduce the frequency of surgery [10-12] and improve cosmetic appearance [13-15]. Poor cosmetic appearance for the patient may be the most important problem, which can be solved or at least reduced by the use of advanced bracing techniques including the best possible correction principles available to date [13]. Pattern specific bracing is desirable and it was Rigo [1] who implemented a new classification with 15 different curve patterns. All those curve patterns demand individual principles of correction in 3D, however 5 key patterns have been identified which we can start working with in everyday practice. These key patterns have been included into a guideline for brace construction that can be used for the custom plaster cast technique, for CAD designed braces as well as for the braces constructed via off the shelf construction kits like the "Chêneau light"^® ^brace (Patent pending) [16] developed recently.

Aim of this new development was to make the brace lighter, finer, easier to wear, and by this to allow a better quality of life for the patients with scoliosis under brace treatment.

This is accomplished by using less material in comparison to traditional bracing systems, which are intended for scoliosis treatment (Fig. 1 and 2). However material reduction should not result in reduced effectiveness. Since scoliosis corrective bracing treatment depends on the primary correction effect achieved in the brace [17-19], the primary correction effect in the „Chêneau light"^® ^brace has been evaluated and compared with that of other braces used traditionally.

**Figure 1:**
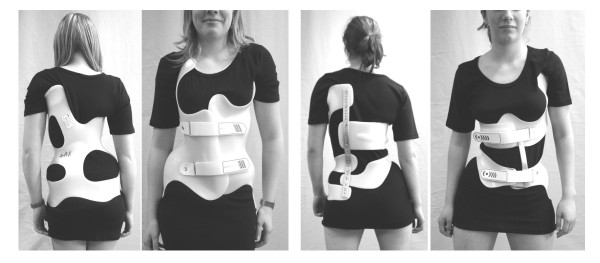
**Comparison Chêneau/Chêneau light**. Dorsal and ventral aspect of two patients with comparable curve patterns. The left patient wears a Rigo-System Chêneau (RSC) brace and the patient on the right a Chêneau light brace. The material necessary for the Chêneau light brace is clearly less.

**Figure 2:**
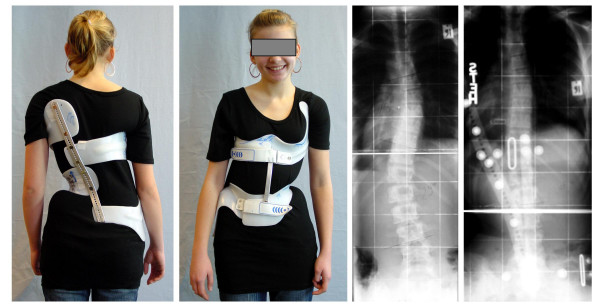
**Example of a double major scoliosis treated with a Chêneau light brace**. Nearly 13 year old girl with a 29/29° Double major AIS in the ScoliOlogiC „Chêneau light" corrected to 11/9° Cobb.

## Methods

From October 2005 on all patients with a "Chêneau light" brace have been listed in a data base in order to allow prospective follow-up studies in the future. The responsibility to list every patient treated with such a brace was taken over by the third author (CS). At the time this paper was prepared there were 132 Patients with an in-brace x-ray in the "Chêneau light" data base, however only 81 fulfilled the criterion to be braced for the first time. 51 Patients had a brace other than a "Chêneau light" before the onset of the treatment with this brace. The braces these patients had prior to the last one, qualitatively showed a wide variety and many of the patients became a "Chêneau light" because they had a progression in the previous brace. This is why these patients have not been included in this study.

The correction effects of the first 81 patients (main diagnosis AIS [n  = 64]or EOS [n = 15]), treated according to the principles of the "Chêneau  light" brace were evaluated after an average treatment time of 6 weeks by a  full-body X-ray taken in the standing position whilst wearing the brace and  compared with the last X-ray before bracing. The average curvature angle of  the whole group was 35,6° (SD 11,5; range 20-69°), the average age was 12,9  years (SD 1,9; range 8-19 years), average Risser sign was 1,3 (SD 1,5; range  0-4), average Tanner rating 2,75 (SD 0,7; range 1-4). Additionally to the  patients with Idiopathic Scoliosis two patients with neuromuscular scoliosis  have been included in this study. One had a neuromuscular impairment of  unknown origin, the other due to cerebral bleeding but both were able to  walk freely.  

The ScoliOlogiC^® ^off the shelf bracing system enables the orthopaedic technician to construct a light brace for scoliosis correction from a variety of pattern specific shells to be connected to an anterior and a posterior upright. This brace is called „Chêneau light" brace. The advantage of this new bracing system is that the brace is available immediately, easy adjustable and that it can also be easily modified. This avoids construction periods of sometimes more than 6 weeks, where the curve may drastically increase during periods of fast growth. The disadvantage of this bracing system is that there is a wide variability of possibilities to arrange the different shells during adjustment. Therefore the technician has to acquire a deep understanding of basic biomechanics, functional diagnosis and curve pattern identification before being able to apply "Chêneau light " braces. Another disadvantage is that due to the mobility of the shells against each other the brace needs more service than the rigid braces used to date. The service intervals should therefore not be longer than 12 weeks.

Shells are available for the treatment of right thoracic and left lumbar curves in three sizes allowing brace adjustments for most of the adolescent patients. For patients with thoracolumbar curve patterns, for left thoracic, right lumbar curve patterns and for smaller sizes a "Chêneau light" brace can be constructed using the plaster cast technique.

## Results

The Cobb angle in the whole sample has been reduced by an average of  16,4° (SD 8,4; range 0-45°), which corresponds to a correction effect of 51%  (SD 36,4; range 0-220°). The average percentage of correction was calculated  on the basis of the individual percentages and not on the basis of the  average degree values. The differences were highly significant in the T-test  (T = 17,4; p < 0,001). The correction effect was highest in lumbar and  thoracolumbar curve pattern (62 %; n = 18). In thoracic scoliosis the  correction effect was 36 % (n = 41) and in double major curve pattern 50 %  (n = 22). For every patient only the biggest of the curves was evaluated  which means for double major curve pattern only one curve is included into  the database and only the correction of this curve was calculated. The major  curve usually is the curve which corrects least.  

The correction effect correlated slightly negative with age (r = -0,24; p = 0,014), negatively with the Risser stage (-0,29; p = 0,0096) and correlated negatively with the Cobb angle measured before treatment (r = -0,43; p = 0,0001).

The correction effects differed from 0% to 220% (from 20° to minus 24° in one case). The patient without any correction had a brace which showed to be maladjusted in the x-ray. The readjustment was performed, however no new x-ray was made. Another patient in this sample only had a correction of 2° which surely is not satisfying. However this brace was adjusted in the right way as has been shown on the x-ray, but the curve seemed too stiff to achieve more at the moment the x-ray was taken.

7 patients were overcorrected (Fig. 3), two of them with a slight phase shift of the curve which means the correction forces did not aim at the apex of the curve. In those cases the brace was also readjusted resulting consequently in a possibly smaller correction after readjustment; however this is also not registered in this data base because no new in-brace x-rays were taken after correction or readjustment of the brace.

**Figure 3:**
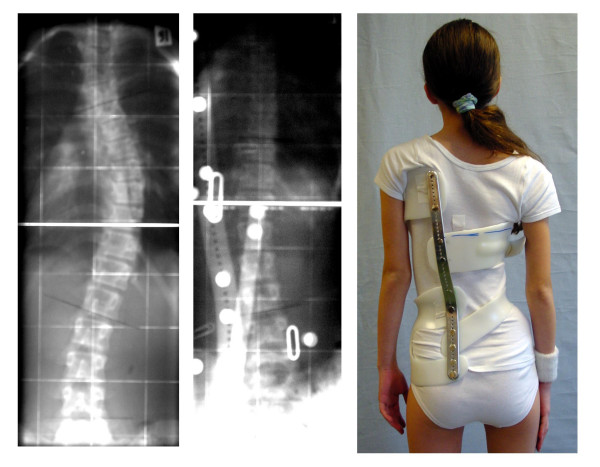
**Example of a patient with an overcorrection in a Chêneau light brace**. Overcorrection of a thoracic curve from 38° to -14° in a T2 „Chêneau light" model in an 11-year old premenstrual girl with Tanner II. For the measurement of the corrected curve the neutral vertebra of this X-ray was used. If we would have taken the neutral vertebrae of the previous X-ray the correction effect would have been less important.

## Discussion

Different bracing concepts are used today for the treatment of scoliosis. The plaster cast method worldwide seems to be the most practiced technique at the moment. CAD systems are on the market which allow brace adjustments without plaster. The latest development however, is the use of the ScoliOlogiC^® ^off the shelf system enabling the orthopaedic technician to construct a light brace for scoliosis correction from a variety of pattern specific shells to be connected to an anterior and a posterior upright designed for full day treatment (Fig. 1, 2, 3, 4).

**Figure 4:**
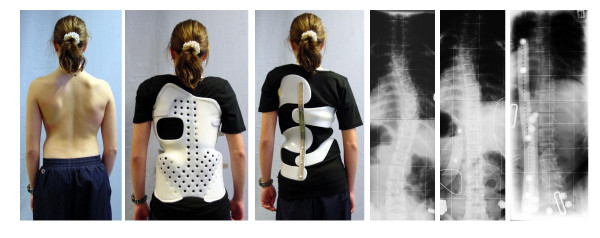
**Example of a patient with two different high correction braces**. 13-year old girl with AIS (39° thoracic). In the previous brace she had 22° high thoracic, 12° low thoracic and 5° lumbar, while in the Chêneau light^® ^brace she has 22° high thoracic, 8° low thoracic and 11° lumbar. The lumbar correction has not been improved after this x-ray in order to achieve a better balance of curves after treatment and a better cosmetic result. The reduction of material in the Chêneau light^® ^brace compared to the previous brace is clearly visible.

After having improved the correction of the braces also in sagittal plane, we were able to improve the correction effect in the frontal plane as well. Compared to the correction effects we achieved 2003 [8], the results now seem significantly better.

In the normal range of brace indications a correction effect of at least 20% is necessary to prevent progression [17], while a correction effect of at average 30% promises some final corrections [18]. A correction effect of 40% and more in a growing adolescent may lead to a final correction of at average 7° Cobb [6].

Wong et al. [19] report correction effects of an average of 40 % in patients with an average Cobb degree of 30,6° (21° – 43°). However in this collective no patients with double curve pattern have been included, which generally corrected worse than single curves in our preliminary study [20].

Bullmann et al. [21] reported average correction effects of 43% in the custom Chêneau brace constructed via plaster cast in patients with a Cobb angle of 31° (25° – 40°). The final rate of success in this study however, was only 58%, which has to be regarded as rather disappointing, when compared to the success rate of 80% we reported on in another study [9] with an average correction effect of less than 40% in custom Chêneau braces constructed via plaster cast.

A modular off the shelf orthopaedic brace for recumbent treatment has been described by Trudell [22]. This so called "bending brace", does not correct in 3D and the shells provided do not allow a proper adjustment for a full-day treatment. A full-day treatment, however, is necessary for a successful end result [23].

Additionally, this brace needs metal connection plates to adjust the shells to the anterior and posterior upright, whilst in the "Chêneau light" brace the shells are connected directly to the uprights giving the system the flexibility needed for the treatment of different curve pattern.

Therefore the "Chêneau light" brace can be regarded an effective tool (possible to be worn full time, good correction effects) for the treatment of adolescents with scoliosis in the majority of the cases. Only certain thoracolumbar curve pattern as well as left thoracic and right lumbar curves need a pattern specific CAD or plaster based construction as long as specific shells are not available to also address those curves.

The data of all patients treated with the "Chêneau light" brace currently are submitted to a database allowing a prospective follow-up of the patients until the end of treatment. An evaluation on quality of life during brace treatment is underway.

Unfortunately, even today, studies with insufficient bracing technologies are published with correction effects of at average less than 25% [24], while the standard in advanced centres is reported to be different for more than 20 years now. In the latter study [24] less than 80% of the population had at least some correction effect in the brace. To irresponsibly apply such poor treatment nowadays can be easily avoided and it needs to be, for our patients deserve a positive effect when they sacrifice their quality of life over years to the prescribed brace in good faith.

## Conclusion

The use of the "Chêneau light" brace leads to correction effects above average when compared to correction effects of other braces described in literature. The reduction of material seems to affect the desired correction in a positive way.

## Competing interests

The first author is currently applying for a patent relating to the content of this paper. None of the authors has received any reimbursements, fees, funding, or salary related to the content of this paper.

## Authors' contributions

HRW: Study design, data acquisition, analysis and interpretation of data, preparation of the manuscript, corresponding author

MW: Data acquisition.

CS: Data acquisition, data base
